# Atrial inflammation and atrial function after cardiac surgery: can we study it properly?

**DOI:** 10.1007/s12471-015-0658-9

**Published:** 2015-01-31

**Authors:** Elton Dudink, Harry J. Crijns

**Affiliations:** Department of Cardiology and CARIM, Maastricht University Medical Center, P Debyelaan 25, 6229HX Maastricht, The Netherlands

Atrial fibrillation (AF) occurs frequently after cardiac surgery and is mostly self-limiting. It relates to direct surgical effects on the atria, atrial ischaemia during surgery, adrenergic activation and a general state of inflammation. Although most postoperative AF is transient, it may lead to a prolonged hospital stay and increased mortality [1]. Treatment strategies include the use of beta blockers, several antiarrhythmic drugs such as propafenone, sotalol and amiodarone, antioxidants to reduce ischaemia-reperfusion damage, angiotensin inhibition and anti-inflammatory medication such as N-acetylcysteine, naproxen, colchicine and glucocorticosteroids.

In this edition of the *Netherlands Heart Journal*, Jacob et al. [2] report a substudy of the DECS study [3], which aims to describe the influence of a single high-dose infusion of the glucocorticoid dexamethasone during cardiac surgery on the perioperative function of the left atrium and the development of postoperative AF.

The authors have put much effort into the sophisticated analysis of transoesophageal echocardiography recordings (TEE). We commend the investigators for their focus on atrial function rather than atrial size only, especially since atrial function may better reflect atrial pathophysiology than merely looking at atrial anatomy. Their methodology should be followed when evaluating atrial performance in order to understand atrial remodelling processes much more efficiently.

In the original DECS study [3], dexamethasone did not reduce the incidence of postoperative AF, suggesting that a single dose of dexamethasone cannot effectively ameliorate atrial inflammation, which is one of the presumed arrhythmogenic mechanisms. In line with that previous observation in the main DECS study, the present substudy shows that dexamethasone is not associated with preservation of atrial function at the very end of the operation.

We again commend the investigators for having performed such extensive TEE analyses. We understand that collecting data immediately after surgery was most appropriate considering the complex logistics and limited acceptance of TEE in the awake patient. On the other hand, atrial inflammation may not be at its peak right after surgery and therefore the investigators may have missed the beneficial effects of dexamethasone on atrial function. This notion is fed by the fact that postoperative AF reaches its highest incidence on day two after cardiac surgery [4].

Comparing preoperative and postoperative left atrial (LA) function within the dexamethasone and placebo groups (rather than the presented comparison between dexamethasone and placebo before and after surgery) suggests that the LA total emptying fraction is stable in the placebo group, while it seems to decrease in the dexamethasone group. Whilst correction of the LA function parameters for fluid status may appear explanatory one cannot exclude an early unexpected deleterious effect of dexamethasone potentially precluding a net positive effect during the next few days after surgery. Future studies on postoperative atrial inflammation may shed more light on these notions.

The present study shows the feasibility of assessing perioperative atrial function using TEE. However, it also illustrates the limitations of fitting methodology to standard care which precluded observing atrial inflammation when it—expectedly—was at its peak. To paraphrase the present Dean and Vice Chairman of the Board of the University Medical Centre Utrecht, it is time for a ‘transition in science’ [5]. Among others, this may be taken by clinical scientists as a call for always creating the most optimal methodologies whilst respecting clinical routines. This certainly will contribute to producing high-quality papers with impact, which can be used to guide important clinical decisions and thus improve the quality and length of patients’ lives [5].

## Funding

None.

## Conflict of interest

None declared.
